# Age-Adjusted Associations Between Comorbidity and Outcomes of COVID-19: A Review of the Evidence From the Early Stages of the Pandemic

**DOI:** 10.3389/fpubh.2021.584182

**Published:** 2021-08-06

**Authors:** Kate E. Mason, Gillian Maudsley, Philip McHale, Andy Pennington, Jennifer Day, Ben Barr

**Affiliations:** Department of Public Health, Policy and Systems, University of Liverpool, Liverpool, United Kingdom

**Keywords:** COVID-19, coronavirus, comorbidity, chronic disease, review

## Abstract

**Objectives:** Early in the COVID-19 pandemic, people with underlying comorbidities were overrepresented in hospitalised cases of COVID-19, but the relationship between comorbidity and COVID-19 outcomes was complicated by potential confounding by age. This review therefore sought to characterise the international evidence base available in the early stages of the pandemic on the association between comorbidities and progression to severe disease, critical care, or death, after accounting for age, among hospitalised patients with COVID-19.

**Methods:** We conducted a rapid, comprehensive review of the literature (to 14 May 2020), to assess the international evidence on the age-adjusted association between comorbidities and severe COVID-19 progression or death, among hospitalised COVID-19 patients – the only population for whom studies were available at that time.

**Results:** After screening 1,100 studies, we identified 14 eligible for inclusion. Overall, evidence for obesity and cancer increasing risk of severe disease or death was most consistent. Most studies found that having at least one of obesity, diabetes mellitus, hypertension, heart disease, cancer, or chronic lung disease was significantly associated with worse outcomes following hospitalisation. Associations were more consistent for mortality than other outcomes. Increasing numbers of comorbidities and obesity both showed a dose-response relationship. Quality and reporting were suboptimal in these rapidly conducted studies, and there was a clear need for additional studies using population-based samples.

**Conclusions:** This review summarises the most robust evidence on this topic that was available in the first few months of the pandemic. It was clear at this early stage that COVID-19 would go on to exacerbate existing health inequalities unless actions were taken to reduce pre-existing vulnerabilities and target control measures to protect groups with chronic health conditions.

## Introduction

After first emerging at the end of 2019, the novel coronavirus SARS-CoV-2 had infected at least 4.6 million people globally by mid-May 2020, with 0.3 million deaths. By July 2021, this had increased to at least 178.8 million people infected and 3.8 million deaths (1). The pandemic remains uncontained in many parts of the world, raising grave concerns about vaccine distribution keeping pace with subsequent waves and new variants. To minimise mortality and morbidity as the pandemic continues, and to direct scarce resources most appropriately, it is crucial to understand better the risk factors for progression to severe coronavirus disease 2019 (COVID-19) and death. It is also important to capture the nature of the evidence that was available to policymakers in the early months of the pandemic, especially given the many government enquiries that will be undertaken around the world into how well the early response tackled the crisis.

Initial reports from China and Italy suggested that people with underlying comorbidities were overrepresented in hospitalised cases and were at increased risk of progression to severe disease and death (2–4). Other countries subsequently reported similar findings (5, 6). Given that the prevalence of comorbidity increases with age, it was unclear whether and how comorbidity independently influences COVID-19 outcomes. Many early studies of COVID-19 epidemiology reported baseline comorbidities of hospitalised patients but not age-adjusted estimates of excess risk associated with comorbidities. Given the high prevalence of chronic disease globally (7), a better understanding of the age-adjusted relationship between comorbidity and COVID-19 outcomes would enhance health service planning and inform clinical management.

We conducted a rapid but comprehensive review of studies in the early stages of the pandemic when hospitalised patients were the population subgroup most readily accessible for research. We are mindful, however, of potential selection bias in these samples due to differential healthcare use, limited SARS-CoV-2 testing in the wider population, and under-ascertainment of asymptomatic and mild cases (8). This review therefore sought to characterise the international evidence base available in the early stages of the pandemic on the association between comorbidities and progression to severe disease, critical care, or death, after accounting for age, among hospitalised patients with COVID-19. We considered evidence to mid-May 2020, 5 months after the viral infection was first identified in Wuhan, China, and only 2 months after the World Health Organization (WHO) declared it a pandemic.

## Methods

### Search Strategy and Selection Criteria

Our search was designed to address the question: according to the evidence base in the early stages of the COVID-19 pandemic, what was the age-adjusted association between co-morbidities and severe outcomes in hospital patients? We searched the literature to identify age-adjusted estimates of association between any comorbidity and in-hospital severe COVID-19 outcomes (Table 1), reported in peer-reviewed studies, pre-prints from repositories such as medRxiv, and several grey literature sources, published by 14 May 2020, in English, from any country (some searches preceded this date; see below). We defined comorbidity as a pre-existing health condition present at admission to hospital with COVID-19, including obesity but excluding health-related behaviours such as smoking.

**Table 1:** Review inclusion and exclusion criteria: what was the age-adjusted association between comorbidities and severe or critical care outcomes in hospital patients with COVID-19 in the early stages of the pandemic?

	**Include**	**Exclude**
Population	Adult COVID-19 hospital patients. Studies with 10 or more patients.	Samples nested in clinical trials, samples from cruise ships, familial clusters. Community cases not receiving care in hospitals, including general population estimates of the spread of COVID-19. Studies focusing solely on infants and children (not part of a study including adults).
Outcomes	Relative risk, hazard ratio, odds ratio associated with comorbidity (pre-existing condition, chronic illness) status on admission, of: i. progression to severe disease ii. admission to critical or intensive care unit iii. invasive or non-invasive ventilation iv. death in hospital v. any adverse event (i.e., composite indicators of any of i–iv), for any reported comorbidity.	Other treatments inside and outside critical or intensive care departments, e.g., rates of patients receiving oxygen supplementation.
Comparison	Patients with and without any comorbidity at admission to hospital. Comorbidity was defined as pre-existing health conditions present at admission to hospital with COVID-19, including obesity.	Comparisons within a sample of patients who all have a comorbidity (e.g., studies of cancer patients only). Comparisons between groups of people based on their health-related behaviours (e.g., smoking), ethnicity, or socioeconomic circumstances.
Study design	All primary quantitative empirical observational studies that reported estimates of the independent relative hazard/odds of experiencing a severe outcome *according* to comorbidity status, adjusted for age only, or age and other plausible confounders of that association.	Any studies in which all estimates of excess risk associated with comorbidity were also adjusted for potential mediators between comorbidity and severe disease outcomes, such as clinically ascertained biomarkers (e.g., inflammatory response or organ function). Causal interpretation of hazard/odds ratios is inappropriate from models not designed to account for confounding of the exposure-outcome association of interest (9, 10), therefore in this review estimates would likely be biased towards the null if adjusted for clinical biomarkers. Qualitative studies. Intervention studies (e.g., clinical trials of new treatments for COVID-19). Projections or estimations of potential outcomes. Non-empirical studies, including editorials, opinions, or discussion pieces. Studies that do not report comorbidity-related risk estimates Review-level evidence
	**Include**	**Exclude**
**Publication characteristics**		
Publication stage, type	Pre-prints, peer-reviewed publications, grey literature on empirical evidence (e.g., official statistics).	Not applicable.
Language	English language publications.	Non-English language publications (not available for full text).
Date	Studies published between December 2019 and 14th May 2020.	

**Figure 1:**
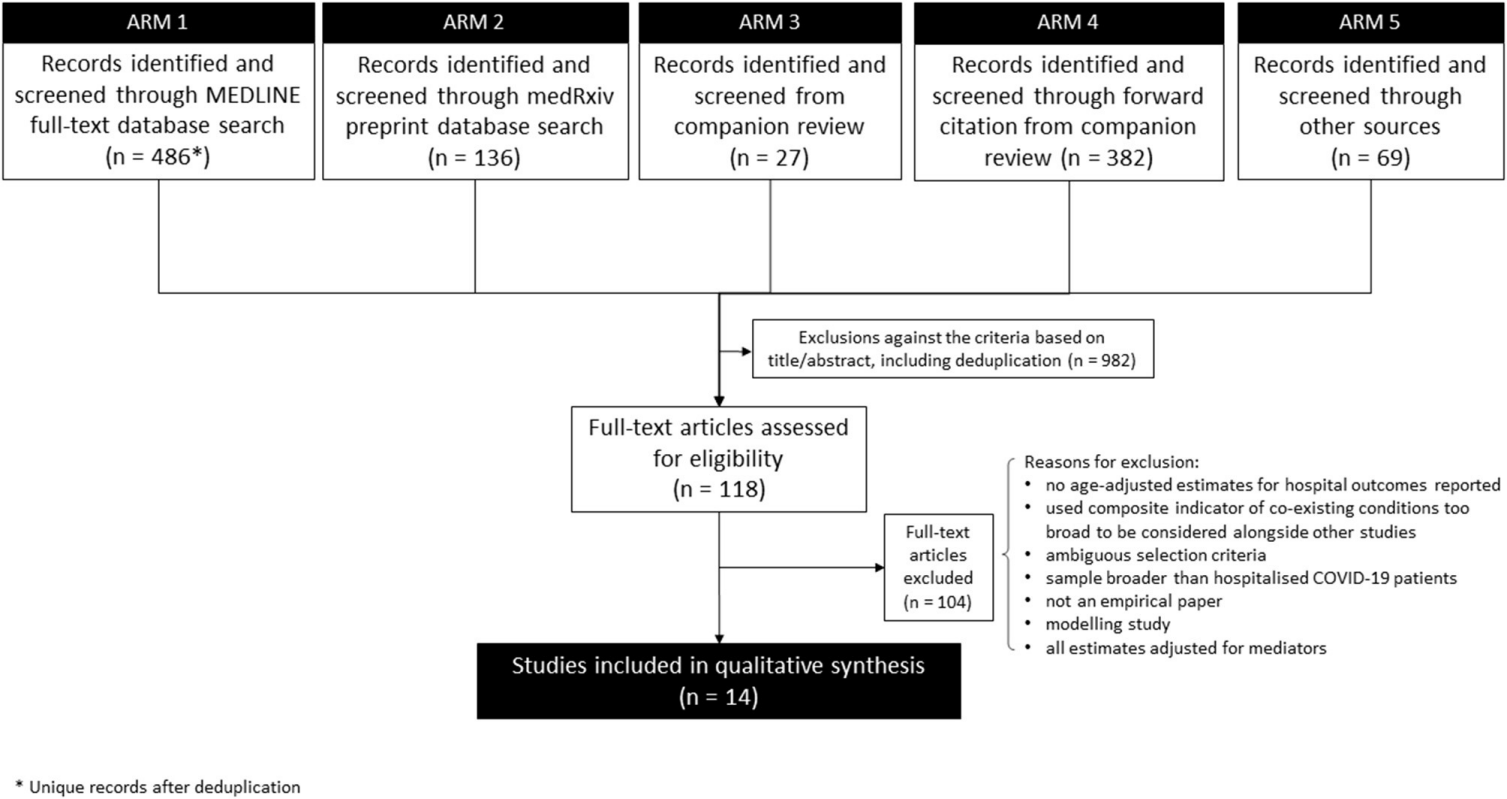
Flowchart of progression of studies through the review of age-adjusted associations between comorbidity and outcomes of COVID-19 in the early stages of the pandemic.

The search strategy had five arms (Figure 1). First (7 April; updated 12 May 2020), we searched the MEDLINE full-text database (as title and abstract often omit age-adjustment) to identify analytical (rather than descriptive) studies that focused on comorbidities specifically or that reported multivariable analysis of risk factors (including comorbidities) for severe outcomes of COVID-19 (see Appendix 1 in Supplementary Material for full search terms). Second (on 14 May), we searched the medRxiv pre-print database. Third, we screened studies from our companion review on COVID-19 critical care outcomes (Pennington et al. unpublished, which by then had screened 2,665 items) for any meeting our narrower search criteria. Fourth, all studies identified in that companion review underwent Web of Science and Google Scholar forward-citation searches, with initial filtering for key terms relating to comorbidity and age (on 7 April). Fifth, additional sources searched (initially in April; updated 11 May) included: WHO; communicable disease centres of the USA, Europe, and China; and several COVID-19-specific evidence resources online (“other sources” in Figure 1; see Appendix 1 in Supplementary Material for details).

### Screening for Inclusion

In Arms 1 and 2, title-abstract screening by one reviewer excluded studies clearly not meeting the inclusion criteria, followed by independent title-abstract screening of the remainder by two reviewers in EPPI Reviewer-4 (11). In each of Arms 3–5, title-abstract screening was followed by full-text screening by a single reviewer. Outputs of Arms 1–5 were combined, and remaining duplicates were excluded. To facilitate this *rapid* review, three reviewers shared searching and screening tasks, rather than repeating tasks independently, except where otherwise stated. Two reviewers independently screened the full text of the final set of potentially eligible studies. On 27 May, included pre-prints were checked for subsequent peer-reviewed publication (and again a year later for the post-script of this definitive article).

### Data Extraction

One reviewer extracted age-adjusted estimates of excess risk (odds ratios or hazard ratios) associated with any comorbidity for the outcomes of interest. A second reviewer checked each extraction for accuracy. Where studies reported multiple estimates adjusted for different sets of covariates (e.g., age alone, age plus sex), one reviewer extracted all estimates then, with checking by others, selected the most appropriate estimate for reporting in the review, prioritising the most appropriate adjustment, e.g., for age and sex rather than age alone and not including potential mediators.

### Synthesis

Evidence was synthesised narratively (12, 13) and, after piloting, study quality was assessed using a modified version of the Institute of Health Economics (14) quality appraisal checklist for case series studies (as recommended by the National Institute of Health and Care Excellence). Limited numbers of studies assessing each individual comorbidity, heterogeneity in key measures and statistical methods, and the inclusion of non-peer-reviewed pre-prints meant that meta-analysis was inappropriate. Extracted estimates are summarised in forest plots. Studies of mortality reported a mix of hazard ratios (HRs) and odds ratios (ORs), so we present both in the forest plots, but it should be noted that ORs and HRs are not directly comparable – ORs overestimate the relative risk of an outcome in studies (such as these) where the outcome is common (15).

## Results

Overall, 1,100 titles-abstracts and 118 full texts were screened (see Figure 1 for exclusions). Of those 118 full texts, 101 were identified in the MEDLINE search, nine from medRxiv, three from the forward citation search from the companion review, and five from other sources. After full-text screening, 14 studies (16–29) met the inclusion criteria. Of these, seven were published in peer-reviewed journals and seven were identified in non-peer-reviewed pre-print form. Four of the pre-prints were included in the review as pre-prints (19, 25, 26, 29) while three were replaced with a peer-reviewed version before analysis (16, 18, 24).

### Characteristics of the Included Studies

Six of the included studies were from China during the initial stages of the pandemic. Three of these used data from Wuhan and the Hubei province, the epicentre of the Chinese outbreak (17, 27, 29), while the others focused on cases hospitalised outside Hubei, using either national samples (20), city-wide reporting systems (28), or records from a single tertiary hospital in another province (16). Four studies used data from the USA (19, 21, 22, 24), three from the UK (18, 25, 26), and one from Iran (23). Eight were multi-centre studies. Sample sizes for the relevant estimates ranged from 103 (21) to 15,194 (18). Study quality varied from low (quality score 8/20) to moderately high (15/20) (Table 2; Supplementary Figure 1). The narrative synthesis of results focuses more on larger and higher quality studies.

**Table 2:** Summary of included studies in review of age-adjusted associations between comorbidity and outcomes of COVID-19 in the early stages of the pandemic.

**References**	**Comorbidities analysed**	**Setting**	**Single- or multi- centre study**	**Sample size**	**Quality score (max. 20)**
Sapey et al. (25)	Any (of hypertension, diabetes mellitus, cancer, chronic lung disease, and others)	Birmingham, UK	Multi	2,217	15
Docherty et al. (18)	Obesity, hypertension, heart disease, diabetes, cancer, chronic lung disease (non-asthma), and others	UK (nationwide)	Multi	15,194	14
Cai et al. (16)	Obesity	Shenzhen, Guangdong Province, China	Single	387	14
Wang et al. (27)	Hypertension, heart disease	Wuhan, Hubei Province, China	Single	296	14
Palaiodimos et al. (24)	Obesity	New York, USA	Single	200	14
Guan et al. (20)	Hypertension, diabetes, cancer, chronic obstructive pulmonary disease; and any in combination	China (nationwide)	Multi	1,590	13
Zhang et al. (29)	Diabetes mellitus	Wuhan, Hubei Province, China	Single	258	13
Kalligeros et al. (21)	Obesity, hypertension, heart disease, diabetes, lung disease	Rhode Island, USA	Multi	103	13
Teo et al. (26)	Hypertension, ischaemic heart disease, diabetes mellitus	London, UK	Multi	437	12
Ebinger et al. (19)	Obesity, hypertension, heart disease, diabetes mellitus, chronic obstructive pulmonary disease or asthma; and any in combination	Los Angeles, USA	Multi	214	12
Dai et al. (17)	Cancer	Hubei Province, China	Multi	105 cancer patients, 536 controls	11
Nikpouraghdam et al. (23)	Any (of hypertension, cardiovascular disease, diabetes, cancer, chronic lung disease, and other)	Tehran, Iran	Single	2,964	10
Mehta et al. (22)	Cancer	New York, USA	Single	218 cancer patients, 1,090 controls	8
Yu et al. (28)	Hypertension, heart disease, diabetes, lung disease	Shanghai, China	Multi	333	8

Included studies mostly reported retrospective analyses of hospital records, usually with a case series or retrospective cohort study design and assessing associations between multiple risk factors, including comorbidities, and various outcomes. Although many were labelled as cohort studies, they did not generally recruit a random sample (convenience sampling was common), and many did not clarify whether the duration of follow-up allowed all participants to reach a study endpoint or recover. Detail of sample construction in many studies was scant.

For the outcome, three studies reported a composite severe endpoint (all including death or admission to ICU) (17, 20, 26), seven reported death (18, 22–25, 27, 29), and four reported severe disease, including ICU admission (16, 19, 21, 28). Three examined mechanical ventilation or intubation as a separate endpoint (19, 21, 24).

Comorbidities analysed by more than one study were overweight and obesity (five studies), diabetes mellitus (seven studies), hypertension and heart disease (seven studies), cancer (four studies), and chronic respiratory conditions such as chronic obstructive pulmonary disease (COPD) (five studies). The largest study also examined dementia, kidney disease, liver disease, and neurological conditions (not defined but giving stroke as an example). Additionally, four studies reported the association of one of the outcomes with the presence of any (or multiple) comorbid conditions rather than, or as well as, specific conditions (19, 20, 23, 25). Eight studies collated information on comorbidities from medical records, two studies included self-reported comorbidities, and four did not report the data collection method. Two studies reported robust methods of collating data on comorbidities involving cross-checking primary and secondary care records (25) or quality checks on extracted data (19). Only two studies mapped comorbidities to International Classification of Disease (ICD)-10 codes (19, 25).

All studies adjusted for age using multivariable regression models [Cox proportional hazards (*n* = 5), logistic (*n* = 8), both (*n* = 1)]. All but two studies also adjusted for one or more additional covariates, but these differed across studies. Additional covariates used in more than one study included sex, smoking, other specific comorbidities, and ethnicity. Although several papers acknowledged that missingness would probably be substantial in a pandemic context, only four formally reported missing data, and two imputed missing values.

### Comorbidity and Severe COVID-19 Outcomes: Disease Progression, Critical Care, and Mortality

**Figure 2:**
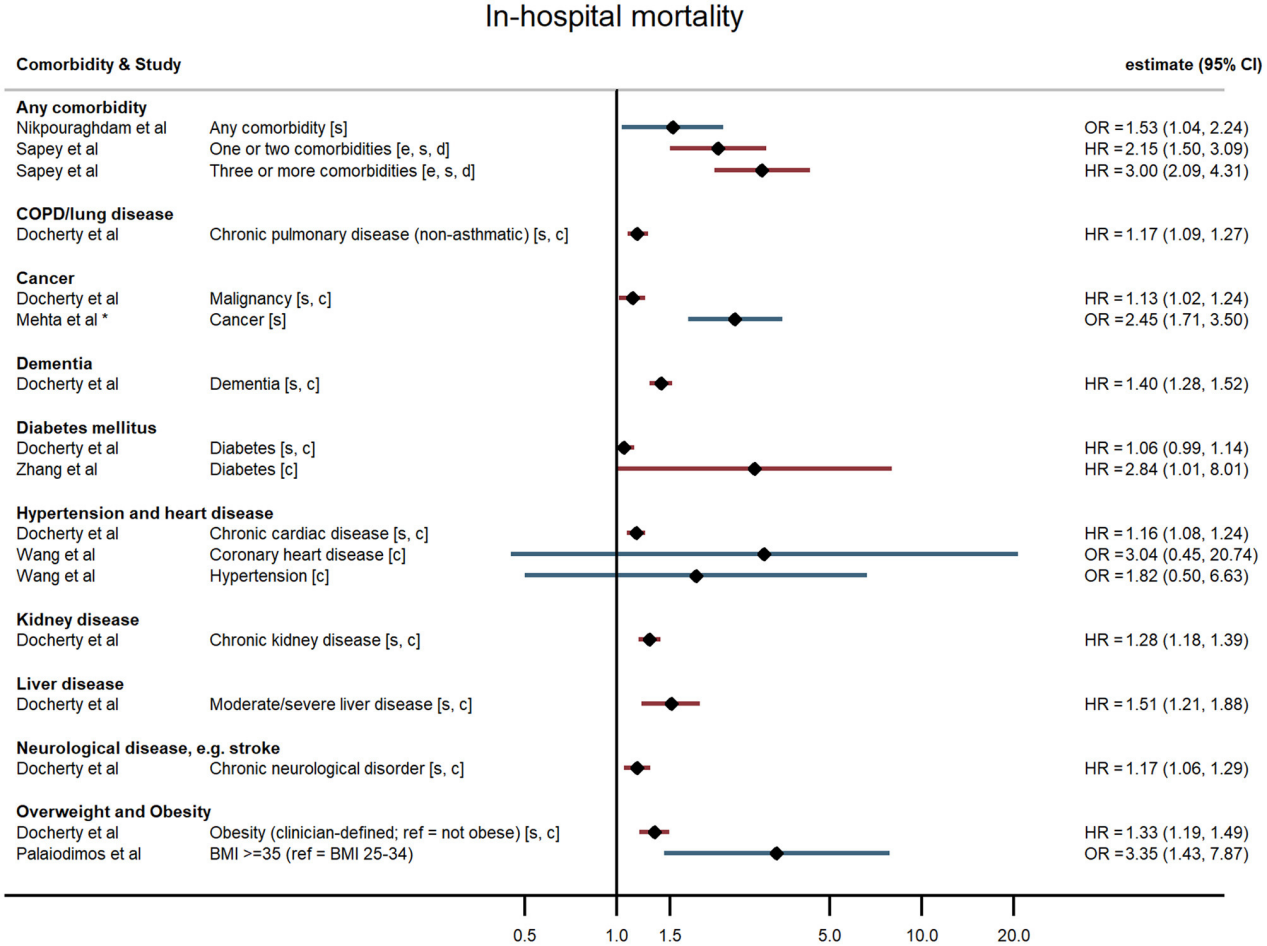
Forest plot of study estimates of the association between comorbidities and COVID-19 mortality among hospitalized patients. Reference category for each comorbidity is the absence of that comorbidity, except where stated otherwise. HR, hazard ratio (red); OR, odds ratio (blue); CI, confidence interval; BMI, body mass index; COPD, chronic obstructive pulmonary disease. All estimates adjusted for age. Additionally adjusted for: sex [s]; other comorbidities [c]; ethnicity [e]; deprivation [d]. *95% CI not reported, but back-calculated from reported *p*-value.

**Figure 3:**
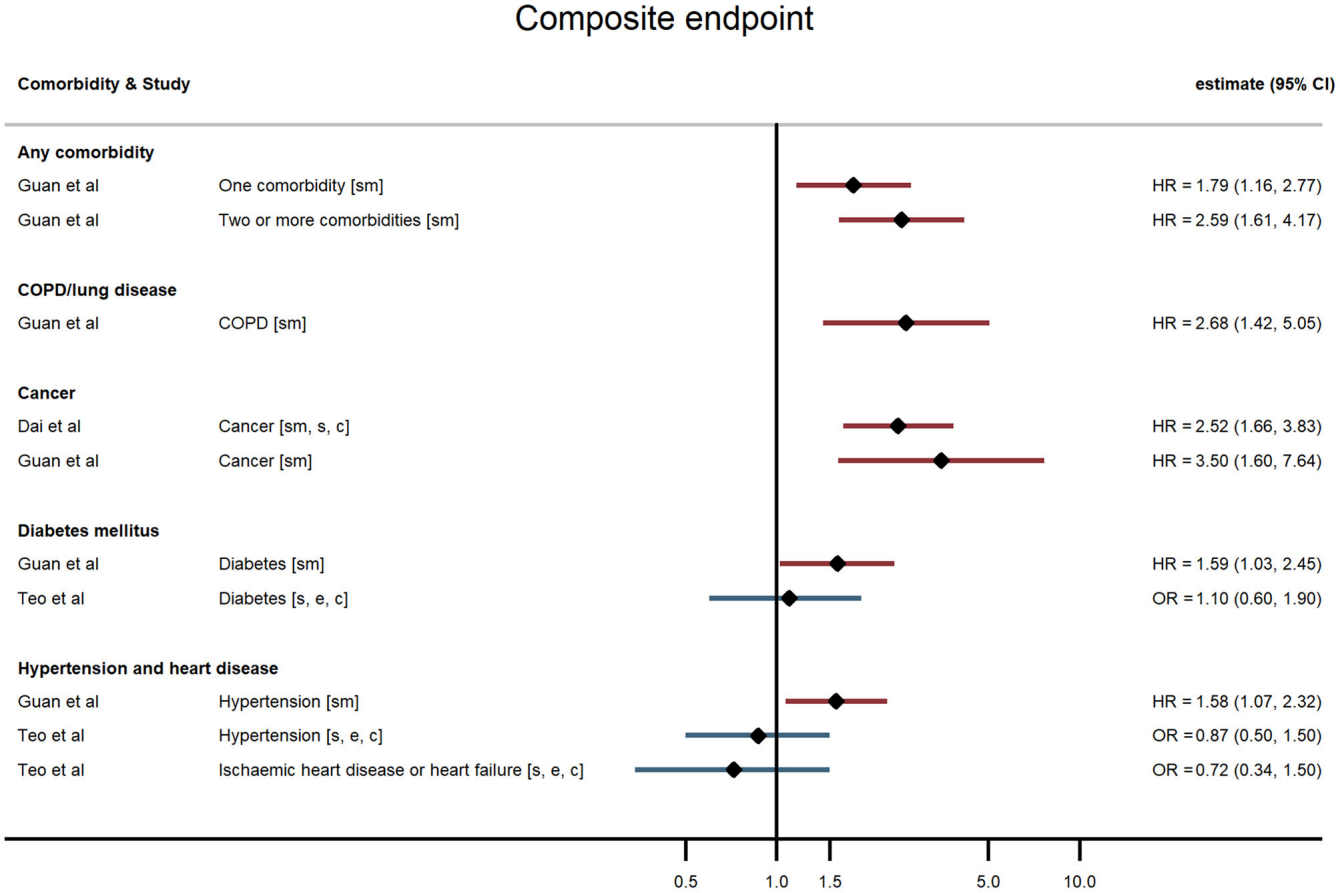
Forest plot of study estimates of the association between comorbidities and composite severe endpoint (death, intensive care unit admission, severe or critical symptoms, or invasive mechanical ventilation) among hospitalized COVID-19 patients in the early stages of the pandemic. Reference category for each comorbidity is the absence of that comorbidity, except where stated otherwise. ICU, intensive care unit; HR, hazard ratio; OR, odds ratio; CI, confidence interval; COPD, chronic obstructive pulmonary disease. All estimates adjusted for age. Additionally adjusted for: smoking [sm]; sex [s]; other comorbidities [c]; ethnicity [e].

**Figure 4:**
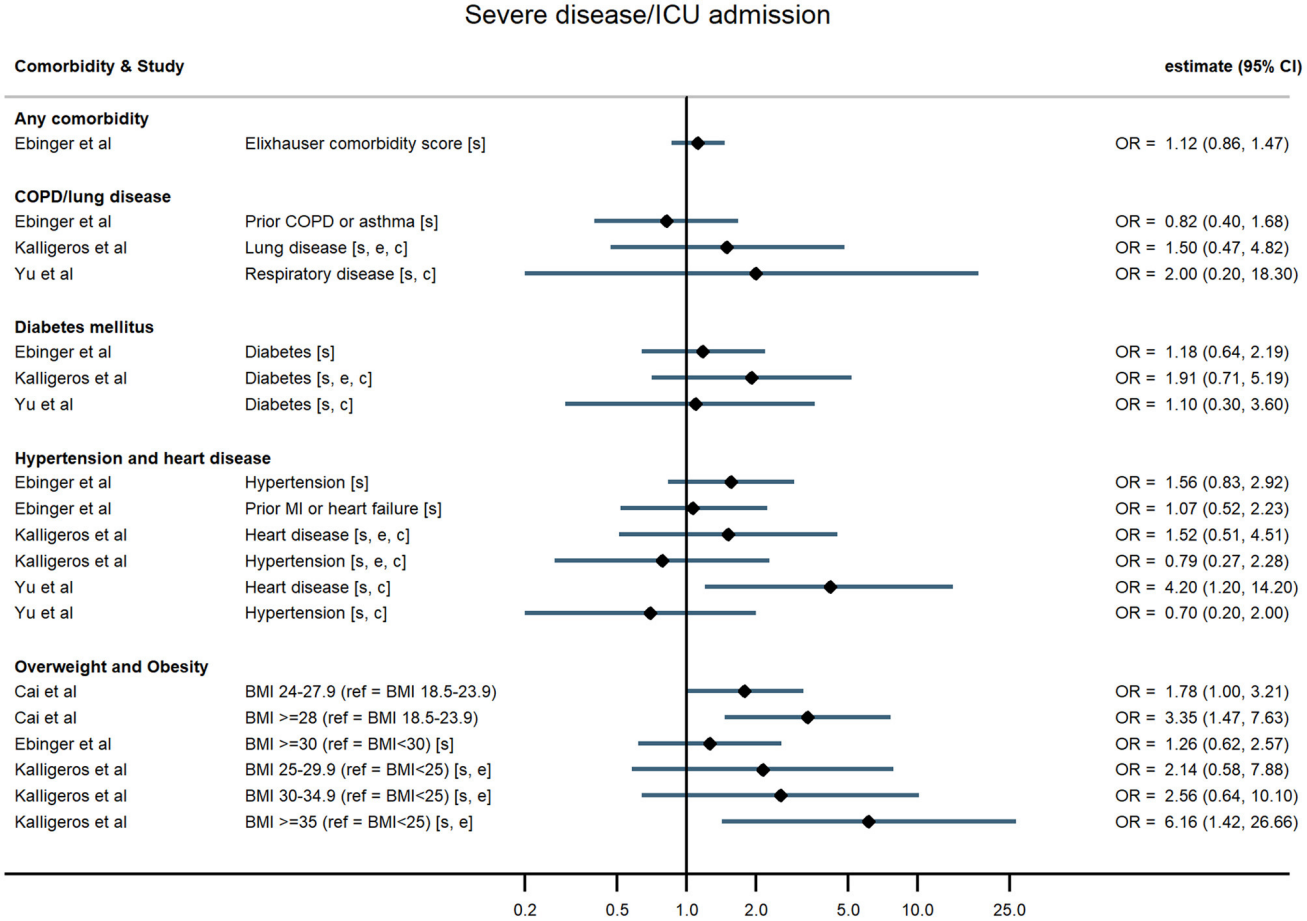
Forest plot of study estimates of the association between comorbidities and progression to severe disease or intensive care unit admission among hospitalized COVID-19 patients in the early stages of the pandemic. Reference category for each comorbidity is the absence of that comorbidity, except where stated otherwise. ICU, intensive care unit; OR, odds ratio; CI, confidence interval; BMI, body mass index; COPD, chronic obstructive pulmonary disease; MI, myocardial infarction. All estimates adjusted for age. Additionally adjusted for: sex [s]; other comorbidities [c]; ethnicity [e].

**Figure 5:**
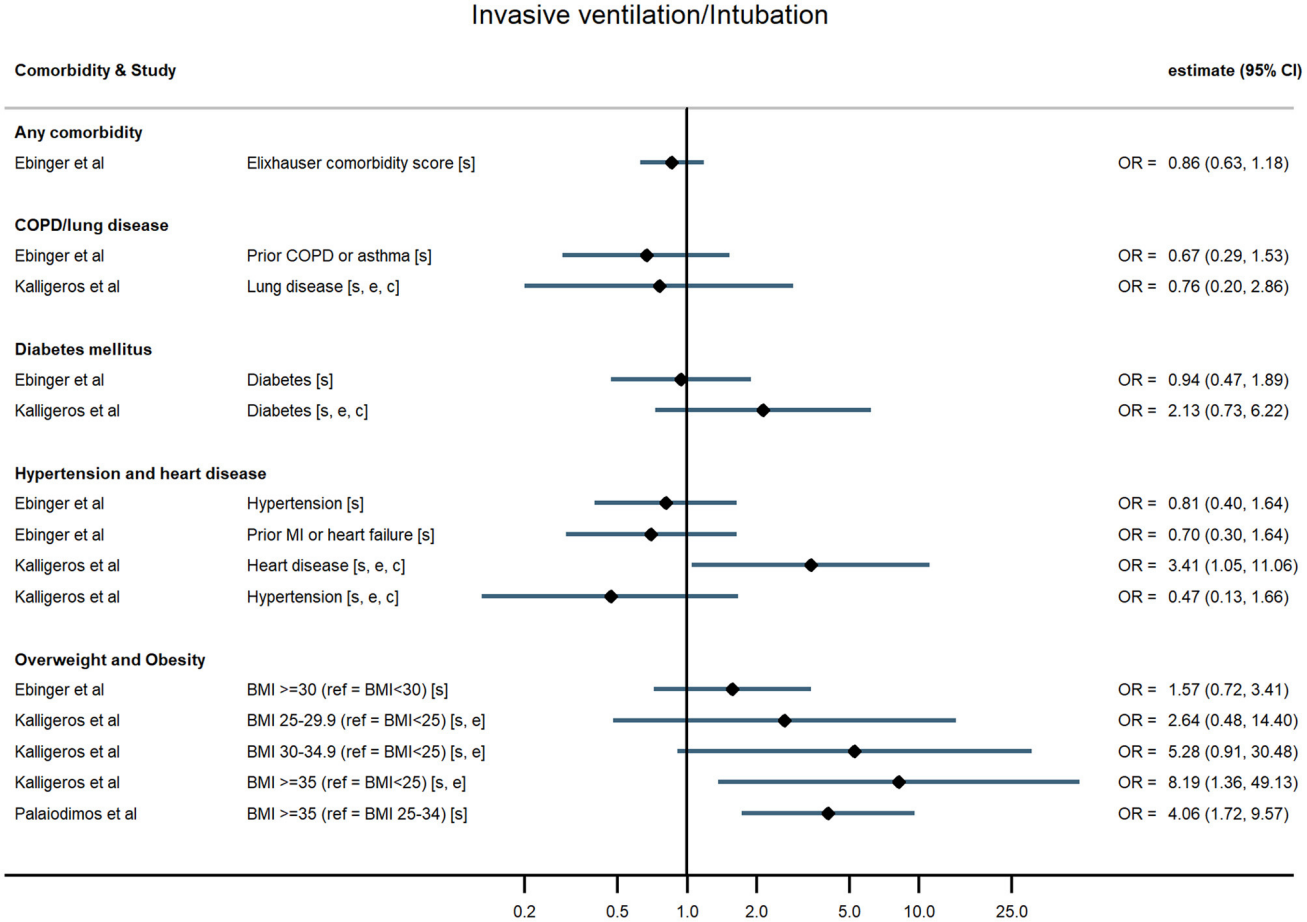
Forest plot of study estimates of the association between comorbidities and invasive mechanical ventilation among hospitalized COVID-19 patients in the early stages of the pandemic. Reference category for each comorbidity is the absence of that comorbidity, except where stated otherwise. OR, odds ratio; CI, confidence interval; BMI, body mass index; COPD, chronic obstructive pulmonary disease; CVD, cardiovascular disease. All estimates adjusted for age. Additionally adjusted for: sex [s]; other comorbidities [c]; ethnicity [e].

Across all comorbidities, the studies with the largest sample sizes and widest geographical coverage consistently showed evidence of increased risk of severe COVID-19 outcomes following hospitalisation associated with the presence of the comorbidity. These associations appeared to hold over studies of varying quality. Smaller studies tended to report similar point estimates to the larger studies but with wide confidence intervals, sometimes consistent with no association, possibly indicating insufficient statistical power. Evidence was most consistent for associations between comorbidities and death but more mixed for other outcomes (Figures 2–5; Supplementary Table 1).

#### Any Comorbidity and Multiple Comorbidities

Hospitalised patients with any comorbidity were more likely to be admitted to ICU, require invasive ventilation, or die from COVID-19, in three of the four studies that examined comorbidities collectively. In a study of 2,217 patients in a large UK city, Sapey et al. examined the effect of “any comorbidity” on mortality and found evidence of a dose response after adjusting for age, sex, ethnicity, and deprivation (25). Patients with one or two comorbidities had a 115% greater hazard of death than patients without comorbidities (95% CI: 1.50, 3.09), and those with three or more had 200% increased hazard of death (95% CI: 2.09, 4.31). Similarly, in a nationwide study of 1,590 patients, Guan et al. (20) also found a dose-response relationship: after adjustment for age and smoking status, patients with a single comorbidity had a 79% greater hazard of a severe outcome than patients without comorbidities, while those with multiple comorbidities had a 159% increased hazard (HR = 2.59, 95% CI: 1.61, 4.17). A study of mortality in 2,964 patients in Iran reported an odds ratio of 1.53 (95% CI: 1.04, 2.24) for any comorbidity compared with none (23). In contrast, a small study in the USA (19) reported on a validated comorbidity score and found no significant association between a standard deviation increase in the comorbidity score and either ICU admission (OR = 1.12, 95% CI: 0.86, 1.47) or intubation (OR = 0.86, 95% CI: 0.63, 1.18).

#### Overweight and Obesity

In the largest study, Docherty et al. (18) reported hazard ratios for various comorbidities and other risk factors among 15,194 COVID-19 patients in UK hospitals. They found that obesity was associated with higher risk of COVID-19 mortality, after adjusting for age, sex, and other comorbidities (HR = 1.33, 95% CI: 1.19, 1.49). In two US studies, severe obesity (BMI ≥35 kg/m^2^) was associated with higher odds of requiring invasive ventilation or dying from COVID-19 (24) compared with people who were overweight or moderately obese, and with higher odds of progressing to severe COVID-19 (21) compared with people of normal weight, independent of age, sex, and ethnicity. Cai et al. (25) examined the relationship between overweight and obesity and progression to severe pneumonia in 387 hospitalised COVID-19 cases in Shenzhen, a Chinese city in Guangdong province. They found that obesity significantly increased the age-adjusted risk of COVID-19 patients developing severe pneumonia (OR = 3.35, 95% CI: 1.47, 7.63). A dose-response relationship was observed, with overweight patients at intermediate risk relative to patients of healthy weight (OR = 1.78, 95% CI: 1.00, 3.21). This relationship was particularly pronounced in men (OR = 5.40, 95% CI 1.93, 15.09). In contrast, a study of 214 COVID-19 patients in Los Angeles, USA, found no evidence of an association between either overweight or obesity and severe disease, or the need for invasive ventilation, having adjusted for age and sex (19).

#### Hypertension and Heart Disease

Evidence of a relationship between pre-existing hypertension or heart disease and severe COVID-19 outcomes was mixed. In China, Guan et al. found that, after adjusting for age and smoking status, patients with hypertension at admission were 58% more likely to reach the composite severe endpoint (ICU admission, invasive ventilation, or death) than those without hypertension (HR = 1.58, 95% CI: 1.07, 2.32) (15). All other studies that examined hypertension (in the UK, China, and the USA) found no evidence of an association with any outcome (19, 21, 26–28).

For chronic heart disease, Docherty et al. (18) found pre-existing disease was associated with higher risk of mortality after adjusting for age, sex, and other comorbidities (HR = 1.16, 95% CI: 1.08, 1.24). Evidence from other studies was mixed. Kalligeros et al. for example, found heart disease to be associated with greater odds of the need for invasive ventilation but not with admission to intensive care (21).

#### Diabetes Mellitus

In studies examining diabetes mellitus as a comorbidity, authors did not distinguish between Type 1 and Type 2 diabetes. The large UK study of 15,194 patients reported a small (6%) but non-significant increased hazard of death for people with compared with those without diabetes following hospitalisation with COVID-19 after adjusting for age, sex, and other comorbidities (HR = 1.06, 95% CI: 0.99, 1.14) (18). The nationwide study in China (20) found that hospitalised COVID-19 patients with diabetes had a 59% increased risk of the composite severe endpoint (ICU admission, invasive ventilation, or death) (HR = 1.59, 95% CI: 1.03, 2.45), independent of age and smoking status. Similarly, in a study of 258 COVID-19 patients at a Wuhan hospital, those patients with diabetes were more likely to die in hospital HR = 2.84, 95% CI: 1.01, 8.01) (29). In contrast though, four other studies found no evidence of an age-adjusted association between diabetes and any severe outcome (19, 21, 26, 28).

#### Cancer

All four studies examining cancer found it to be a risk factor for severe outcomes following hospitalisation with COVID-19. Docherty et al. (18) found a slightly elevated risk of death amongst UK-based COVID-19 patients with cancer (HR = 1.13, 95% CI: 1.02, 1.24). Guan et al. (20) found a substantially elevated risk of their composite severe endpoints – after adjusting for age and smoking status, patients with cancer had 3.5-fold the hazard of ICU admission, invasive ventilation, or death in hospital compared with patients without cancer (95% CI: 1.60, 7.64). Dai et al. (17) found a similar relationship in their sample of cancer patients and matched non-cancer patients in Hubei province, China (HR = 2.52, 95% CI: 1.66, 3.83) after additionally adjusting for other comorbidities. They also reported relative hazards by cancer stage and type, finding that association with the composite severe endpoint was strongest for metastatic cancer and for lung and blood malignancies. Mehta et al. (22) used a similar study design and reported an age- and sex-adjusted odds ratio of 2.45 for cancer and in-hospital mortality.

#### Chronic Obstructive Pulmonary Disease

Five studies reported associations with pre-existing lung disease, either COPD specifically (20) or a broader set of pulmonary conditions (18, 21, 25, 28). One study excluded asthma, two included asthma, and one study did not state whether asthma was included. Docherty et al. reported 17% increased hospital mortality associated with non-asthmatic pulmonary disease (HR = 1.17, 95% CI: 1.09, 1.27), independent of age, sex, and other comorbidities (18). Guan et al. found that hospitalised patients with COPD had 168% higher risk of reaching that study's composite severe endpoint (ICU admission, invasive ventilation, or death) than patients without COPD (HR = 2.68, 95% CI: 1.42, 5.05), adjusted for age and smoking status (20). None of the three remaining studies – using endpoints of progression to severe disease and invasive ventilation – found an association with chronic lung disease, broadly defined (19, 21, 28).

#### Other Comorbidities

One large UK-wide study also found neurological disorders (giving the example of stroke), dementia, liver disease, and chronic kidney disease were all associated with increased risk of mortality after adjusting for age, sex, and other comorbidities (18).

**Post-script:** Of the four pre-prints included in the analysis (19, 25, 26, 29), a final check in May 2021 found that two (19, 25) now had a peer-reviewed publication (30, 31). There were no substantive changes in reported evidence in the published versions compared with the pre-print versions.

## Discussion

### Summary of Findings

Five months after the first outbreak of COVID-19 in Wuhan, China, and 2 months after the WHO declared COVID-19 a pandemic, research was limited on comorbidities as independent risk factors for severe COVID-19. Our review of that emerging evidence base indicates that by mid-May 2020 there was broad support (32) for the hypothesis that many underlying health conditions confer additional risk of mortality among people hospitalised with COVID-19, independent of age. Evidence of increased risk of other severe COVID-19 outcomes was mixed. Most studies found that having at least one of obesity, diabetes, hypertension, heart disease, cancer, or COPD was significantly associated with worse outcomes following hospitalisation. A dose-response relationship was reported for increasing numbers of comorbidities, and evidence linking overweight and obesity to severe outcomes was strongest for more severe obesity. Overall, evidence for obesity and cancer increasing risk of severe disease or death was most consistent, with all but one of the numerous relevant studies reporting an increased risk associated with these conditions. Evidence was weakest for hypertension as an independent risk factor for severe outcomes. Two similar reviews that were published whilst our rapid review was under peer review – and included some more recent studies – found similar results (33, 34), although another found no association with obesity in meta-analysis (33–35).

Comorbidity has previously been shown to be associated with elevated risk of worse clinical outcomes in other severe acute respiratory outbreaks such as SARS (severe acute respiratory syndrome), MERS (Middle East respiratory syndrome), and avian influenza (36–38). This review suggests that comorbidity also pre-disposes individuals to poorer outcomes in the current COVID-19 pandemic. Whilst the mechanisms remain poorly understood, there are numerous biologically plausible explanations. The pathogenesis of severe COVID-19 is thought to involve dysregulated proinflammatory immune response and subsequent multi-system damage (39–41). Many underlying conditions may leave affected individuals more vulnerable to the effects of this. Obesity tends to reduce lung function and dysregulate the immune system (42). Similarly, diabetes mellitus can impair immune function (43), as do many cancer treatments. Patients with pre-existing cardiovascular disease may be at heightened risk of severe outcomes through various mechanisms, including therapeutic upregulation of ACE2 (the host receptor for SARS-CoV-2) and greater vulnerability to hyperinflammatory immune responses and cardiac complications that are common with severe COVID-19 (9, 44).

### Strengths and Limitations of the Evidence Base

This review includes two studies that are still to undergo peer-review. Such studies must be treated cautiously, but the need to summarise timely evidence in an emerging pandemic justifies including them, while requiring “a permanent critical attitude from the readers” (45). Of the other two studies originally included in pre-print form but subsequently peer-reviewed and published within a year of the analysis, we noted no substantive differences between the pre-print and published version. Quality appraisal revealed important limitations in most included studies: weaknesses in design or execution, inadequate detail, or lack of clarity in reporting, particularly around sampling, and inclusion and exclusion criteria.

Our review is limited to the hospitalised population of COVID-19 cases, a highly selected sample of the population. These studies are therefore at risk of selection (or collider) bias (46), which can induce spurious associations leading to flawed conclusions, particularly when the prevalence of a risk factor in the sample differs from its prevalence in the target population (8, 47). Viral load is an unmeasured factor that may differ between hospitalised and non-hospitalised COVID-19 patients and also be associated with risk of severe outcomes. In several included studies, obesity prevalence differed considerably in the study population from the wider population of the same country (16, 18, 19). The extent of missing data was also underreported in many studies, possibly adding further selection bias. The results therefore may not reflect causal effects, and there remains a need for confirmation with larger, population-based studies. Although some studies in this review acknowledged possible selection bias (and it was probably unavoidable early in an outbreak context) none included sensitivity analyses to assess this risk. Furthermore, patients in many studies had not yet reached their clinical endpoint. We now also note that similar reviews were published while ours was under peer review, and are also primarily focused on samples of hospitalised patients [e.g., (33–35)].

There was substantial heterogeneity in outcome and comorbidity definitions and the clarity of their reporting, compromising comparison of results and precluding pooling of estimates. Most studies used electronic health records, but many did not clearly specify data collection methods in any further detail, particularly for recording comorbidities. In some, lack of rigour in comorbidity ascertainment might have led to misclassification, but without more information it is difficult to assess how likely this is as a source of bias. Only two studies included ICD-10 criteria for comorbidities, no study distinguished between Type 1 and Type 2 diabetes, and BMI categorisations also differed between studies.

Across studies, models differed considerably in adjusting for obvious confounders, such as sex or smoking, making comparison challenging. By excluding studies reporting models that contain clinical predictors of disease progression, we excluded hazard ratios or odds ratios for comorbidity that were likely to overadjust for potential mediators (e.g., inflammation) of the possible effect of comorbidity on progression to a severe COVID-19 outcome. We did retain studies that adjusted estimates for one comorbidity by other comorbidities, although this may also lead to overadjustment if one comorbidity mediates another's effect on the outcome (e.g., Type 2 diabetes mediated by obesity). Some of the included estimates are potentially susceptible to residual confounding from omitted confounders such as ethnicity and socioeconomic disadvantage. The estimates in this review do tend, however, to be similar in magnitude across adjustment for various confounders.

Overall, the 14 studies varied considerably in both the quality of the design and reporting, and only a few were moderately high quality. Whilst hasty research and publication are understandable early in such a global emergency, rigour should not be compromised. As London and Kimmelman argued, “the moral mission of research remains the same: to reduce uncertainty and enable caregivers, health systems, and policy-makers to better address individual and public health” (48). Indeed, there is an ethical imperative to ensure that the conduct and reporting of research in a pandemic crisis maintains high standards of validity, reliability, and integrity to provide sufficiently robust evidence for these purposes.

### Strengths and Limitations of the Review

Our review was rapid while also being as comprehensive as possible for that period of the pandemic. A companion review provided forward-citations, and our full-text searches included both pre-print archives and peer-reviewed literature, reflecting the fast-moving early stages of the pandemic and the increasing use of pre-print archives. Full texts were independently screened by two reviewers. To aid interpretation of the synthesised results, we systematically assessed study quality after modifying an existing tool to provide an appropriate appraisal framework for these studies.

The timing of the review meant that almost half the included studies came from China, and the others were restricted to a few other settings. The Chinese studies may have had a healthier case-mix because of different criteria for admission compared with other countries (e.g., the United Kingdom), where only relatively serious cases are hospitalised. The comorbidity profile of China also differs from many other countries (49–51). Our reliance on studies of hospitalised patients means the conclusions only indicate the increased risk associated with comorbidities in hospitalised COVID-19 patients rather than the effect that comorbidities may have on the initial risk of being infected with SARS-CoV-2 or on the outcomes of people with COVID19 outside hospital (e.g., in care homes). As noted above, selection bias may also affect these results. There is also a risk of publication bias - given the short time period, at least some studies published early in the pandemic are likely to have traded quality against the need for timely information. This demonstrates the importance of characterising the early evidence base, so it can later be contrasted will the evidence gathered over the longer run of the pandemic.

The primary focus of this review was to improve understanding of the independent relationships between comorbidities and COVID-19 outcomes, hence we collated evidence from studies where likely confounding by age had been appropriately controlled. With the exception of one meta-analysis that coarsely stratified by age (34), we note that subsequent similar reviews, published while ours was under peer review, have tended not to address explicitly the issue of heterogeneity across studies in terms of whether potential confounding by age was accounted for.

### Implications for Future Data Collection and Research

Early studies will have informed policy decisions in this fast-moving pandemic. Speedy publication in a global emergency necessitated “loosening the critical parameters” (45) for the evidence base, and our quality appraisal suggests that reporting of methods has been adversely affected. Nevertheless, transparent and detailed reporting remains necessary for accurate interpretation. A lesson for future pandemics would be that having pre-agreed international guidelines on consistent methods and reporting of sample selection, description, and design (including variables to be measured and data collection tools) would facilitate more effective use and application of research efforts, by enabling pooling of results from different locations and settings to provide high-quality evidence quickly.

Caniglia et al. (10) argued that science during a pandemic must accommodate unavoidable uncertainty whilst maintaining science's social responsibility to the public good. This would include ensuring better skills, capacity, and theoretical frameworks are in place to enable better mobilisation of evidence under conditions of high uncertainty. Early in the pandemic, hospital-based studies were the most feasible, but population-based studies must now play a larger role in clarifying understanding of various independent risk factors for severe outcomes from COVID-19. Several such studies started emerging from May 2020 onwards [e.g., (52)], but making meaningful inferences can still be challenging (53). Regardless of study design, future studies should attempt to evaluate the robustness of conclusions to plausible sources of selection and other biases.

### Implications for Policy and Practice

As early as May 2020, it was apparent that various comorbidities conferred an increased risk of severe disease progression and death after being hospitalised with COVID-19, independent of age. The evidence was slightly more consistent for obesity, although many other common chronic conditions across organ systems seemed to confer an elevated risk, and there is evidence that multimorbidity adds further risk. Given the relatively high population prevalence of most comorbidities covered in this review, the implications of elevated risk are substantial. It has been estimated from Global Burden of Disease prevalence data that one in five individuals globally may be at increased risk of severe COVID-19 due to underlying conditions (54), but this is likely to underestimate risk because obesity was omitted. Furthermore, this burden is not evenly distributed between countries, meaning COVID-19 is impacting healthcare systems already under pressure from high local burdens of non-communicable disease.

Whether COVID-19 accelerates the underlying condition, or weakened underlying organs or immune response increase vulnerability to severe COVID-19, or both, is subject to ongoing research globally. Nevertheless, even without a full explication of the mechanisms, early epidemiological evidence of an association between comorbidities and poor in-hospital outcomes supported action to protect these groups and mitigate their elevated risk.

The increased risk associated with many comorbidities supported strong, targeted primary prevention measures to “shield” people with comorbidities from SARS-CoV-2 and suggested a need for public health campaigns to promote awareness of these elevated risks and how people could protect themselves. Vaccines have been prioritised for those at higher risk. In terms of secondary prevention of COVID-19, it is important to detect it early in those with comorbidities, to reduce progression as treatments (such as dexamethasone for severe or critical disease) emerge. This evidence has implications for healthcare system demand in areas of high comorbidity prevalence. To address the greater burden of COVID-19 in communities with more pre-existing conditions, greater resources should be allocated according to this need. Approaches in the early stages of the pandemic were, however, not prioritising sufficiently these higher levels of need (55). There are also implications when preparing for subsequent waves of community transmission. In particular, the evidence presented here highlights greater urgency for reducing the prevalence and incidence of chronic disease, through support for non-communicable disease prevention efforts and addressing the wider determinants of health.

Finally, the intersection of underlying comorbidity with socioeconomic disadvantage, geography, and demographic factors, especially ethnicity, has proven to be a potent mix that will widen health inequalities, both within and between countries. In England, official statistics showed that COVID-19 age-standardised mortality rates in the most deprived parts of England are more than double the rate in the least deprived areas (56) and this is partly explained by inequalities in existing chronic health conditions. Furthermore, people from ethnic minority backgrounds are overrepresented among deaths from COVID-19 (57–59), with ethnicity apparently a risk factor independent of deprivation (60), probably partly due to higher prevalence of common comorbidities. In addition, there are numerous social and structural factors that increase risk of infection in these groups (such as overcrowded housing, greater reliance on public transport, and employment in essential and “frontline” occupations with much human contact where physical distancing is not feasible).

People with chronic health conditions are already disadvantaged and underrepresented in the workforce. They have been further disadvantaged by control measures such as prolonged shielding – which can adversely affect their financial, social, and mental well-being. Further use of such measures – in this or another pandemic - will require tailored support and strategies to mitigate impacts.

Without concerted effort, reducing existing risk factors such as obesity and targeting support for people with pre-existing health conditions, the COVID-19 pandemic is likely to widen health inequalities between social, ethnic, and geographical groups. Pandemic responses must therefore prioritise and mitigate the unfair burden shouldered by disadvantaged and ethnic minority groups.

## Conclusions

Building on evidence that people with comorbidities were overrepresented in hospitalised cases of COVID-19, this review compiled estimates from age-adjusted regression models across 14 studies from various settings globally in the early stages of the pandemic. It summarises for clinicians, policymakers, and academics the most robust evidence that was available in those first few months on this topic, to inform decision-making. Characterising this early evidence base helps to provide crucial context for the many enquiries, probes, and reflective exercises that will be performed around the world to scrutinise what should have been done better in the early response. Despite its limitations, the early evidence base showed that people with underlying chronic health conditions are at increased risk of severe disease progression and death and supported a range of public health and clinical approaches to protecting people with comorbidities. Given the distribution of comorbidities in the community, this evidence indicates that COVID-19 will exacerbate existing health inequalities, unless actions are taken to reduce these pre-existing vulnerabilities and target control measures to protect groups with chronic health conditions.

## Author Contributions

BB, AP, KM, and PM conceived of and designed the review. KM, PM, and AP undertook the searching and screening, with search support from the companion COVID-19 critical care review team at the University of Liverpool. KM did the initial data extraction, summarised study characteristics, and led the narrative synthesis. GM checked data extraction for accuracy and missing data. JD completed the quality assessment of the included studies. All authors contributed to the synthesis of evidence and writing of the manuscript.

## Author Disclaimer

The views expressed in this publication are those of the authors and not necessarily those of the NIHR or the Department of Health and Social Care.

## Conflict of Interest

The authors declare that the research was conducted in the absence of any commercial or financial relationships that could be construed as a potential conflict of interest.

## Publisher's Note

All claims expressed in this article are solely those of the authors and do not necessarily represent those of their affiliated organizations, or those of the publisher, the editors and the reviewers. Any product that may be evaluated in this article, or claim that may be made by its manufacturer, is not guaranteed or endorsed by the publisher.
