# Supplementary material for: Age-Adjusted Associations Between Comorbidity and Outcomes of COVID-19: A Review of the Evidence From the Early Stages of the Pandemic
Source: Front Public Health. 2021 Aug 6;9:584182. doi: 10.3389/fpubh.2021.584182 (PMC8377370; doi:10.3389/fpubh.2021.584182)
Supplement: Supplementary file 1 [file Data_Sheet_1.PDF]

SUPPLEMENTARY MATERIAL

Mason KE, McHale P, Pennington A, Maudsley G, Day J, Barr B. Age-adjusted associations between comorbidity and outcomes of COVID-19: a review of the evidence.

S1. Search strategy: additional details

MEDLINE Full Text Search strategy for review of age-adjusted associations between comorbidity and outcomes of COVID-19

MEDLINE with Full Text 12-05-20 Via EBSCOhost, limited to English Language (Dec 2019-May 2020)

> TI ((“Wuhan coronavirus” OR COVID19 OR COVID-19 OR COVID-2019 OR COVID 19 OR COVID 2019 OR “coronavirus disease 2019” OR SARS-CoV-2 OR SARS2 OR 2019-nCoV OR “novel coronavirus” OR “severe acute respiratory syndrome coronavirus 2” OR “severe acute respiratory syndrome coronavirus-2” OR coronavirus-2 OR “coronavirus disease-19” OR coronavirus disease-19 OR SARS-CoV-2019 OR SARS-CoV-19)) AND TX ((mortality OR mortalities OR fatality OR fatalities OR death OR ventilation OR ventilated OR “clinical outcome” “clinical characteristic” OR ICU OR “critical care” OR “intensive care” OR intubated OR intubation OR “hospital admission” OR “admitted to hospital” OR cardio OR cardiac OR heart OR diabetes OR chronic OR COPD OR hypertension OR cancer OR renal OR kidney OR hepatitis OR immunodeficiency OR comorbid* OR underlying OR pre-existing OR pre-existing OR liver OR obesity OR BMI OR severity)) AND TX ((“by age” OR “age adjusted” OR “age specific” OR “age stratified” OR “age standardised” OR “age standardized” OR “age-adjusted” OR “age-specific” OR “adjusting for age” OR “adjustment for age” OR “after adjusting” OR “after adjustment” OR “after-adjusting” OR “after-adjustment” OR multivaria* OR multi-varia*))

Expanders - Apply related words; Also search within the full text of the articles; Apply equivalent subjects

Additional searches of national organisations and repositories

Searched 07/04/2020

- World Health Organization
- Centres for Disease Control and Prevention, USA
- NHS England and NHS Improvement
- Public Health England
- European Centre for Disease Prevention and Control
- Chinese Center for Disease Control and Prevention

Searched 14/04/2020

- Oxford COVID 19 Evidence Service (https://www.cebm.net/covid-19/)
- COVID-19 Scientific Resource Centre: Geneva Centre for Education and Research in Humanitarian Action (https://cerahgeneve.ch/resources/covid-19-free-online-scientific-resources/?_ga=2.226059738.1105476567.1584628506-799341908.1584628506)

S2. Quality appraisal: additional details

Quality of included studies was assessed using a modified version of the Institute of Health Economics quality appraisal checklist for case series studies. This is a tool recommended by the National Institute of Health and Care Excellence for appraisal. While the majority of the studies in this review were retrospective cohort studies, the sampling was in many studies equivalent to a case series design. We piloted alternative quality appraisal tools for cohort studies such as the Newcastle Ottawa scale but they were deemed better suited to large traditional, prospective cohort studies with longer follow-up periods, while we determined that the IHE case series tool – with some modification – was a good match for the acute pandemic studies included in this review.

The original tool can be found here: https://www.ihe.ca/research-programs/rmd/cssqac/cssqac-about

We adapted Items 8 and 9, and 11 (as suggested by IHE) such that:

- "Was the intervention of interest clearly described?" became "Were exposures of interest (i.e. comorbidities) clearly described?" (Item 8)
- "Were additional interventions clearly described?" became "Were additional covariates clearly described?" (Item 9)
- "Were outcome assessors blinded to the intervention that patients received?" became "Were outcome assessors blinded to comorbidity status?".

Item 13 (outcome measurement pre- and post-exposure) and Item 18 "adverse event reporting" were deemed not to be relevant to these observational studies where the outcome was an in-hospital event, and we therefore scored all studies 'Yes' for these items.

Quality appraisal results across the 14 included studies are summarised in Figure S1.

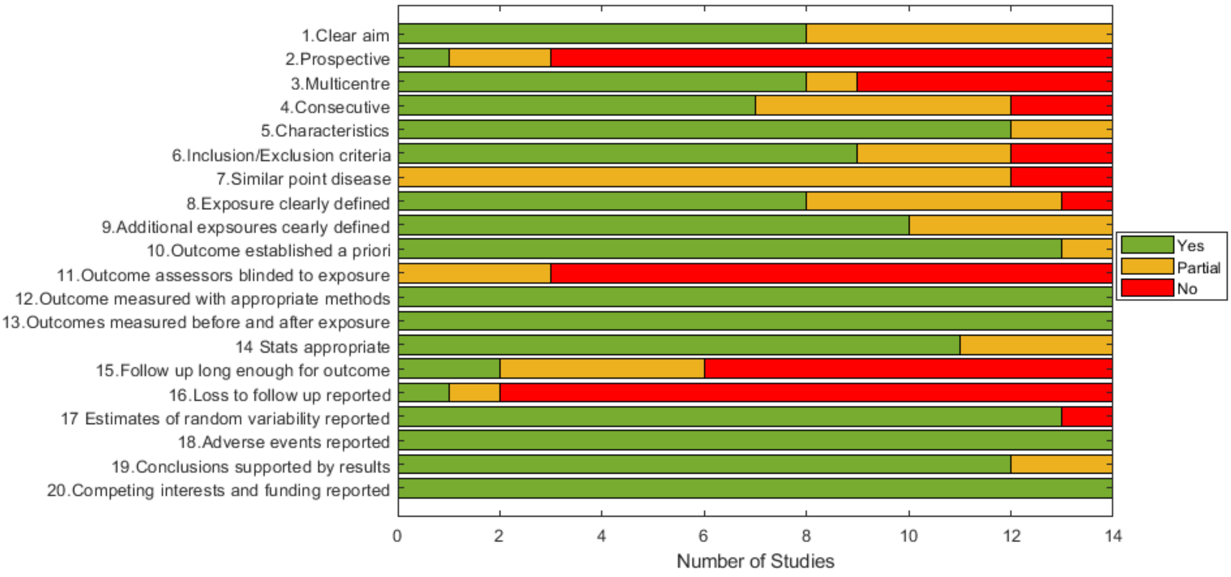

Figure S1. Review of age-adjusted associations between comorbidity and outcomes of COVID-19: Summary of quality appraisal of included studies, based on modified Institute of Health Economics (IHE) tool

S3. Extracted estimates and list of included studies

Table S1: Extracted age-adjusted estimates (and 95% confidence intervals) of excess risk of progression to severe disease or death associated with comorbidities among hospitalised COVID-19 patients (published by 14 May 2020, all from China)

**Comorbidity** (ref=comorbidity not present, except where otherwise stated)	**Composite endpoint: ICU admission, invasive ventilation, or death**	**Death**	**Severe disease (including admission to critical or intensive care unit)**	**Invasive ventilation/Intubation**
Any comorbidity				
One or more		**OR=1.53 (1.04, 2.24) $_{s}$[1]**		
Comorbidity score (Elixhauser; per SD)			OR=1.12 (0.86, 1.47) $_{s}$ [2]	OR=0.86 (0.63, 1.18) $_{s}$ [2]
One only	**HR=1.79 (1.16, 2.77) $_{sm}$ [3]**			
Two or more	**HR=2.59 (1.61, 4.17) $_{sm}$ [3]**			
One or two		**HR=2.15 (1.50, 3.09) $_{e, s, d}$ [4]**		
Three or more		**HR=3.00 (2.09, 4.31) $_{e, s, d}$ [4]**		
Obesity				
Overweight			**OR=1.78 (1.00, 3.21) [5]**	
			OR=2.14 (0.58, 7.88) $_{s, e}$ [6]	OR=2.64 (0.48, 14.40) $_{s, e}$ [6]
Obesity		**HR=1.33 (1.19, 1.49) $_{s, c}$ [7]**	**OR=3.35 (1.47, 7.63) [5]**	
			OR=2.56 (0.64, 10.10) $_{s, e}$ [6]	OR=5.28 (0.91, 30.48) $_{s, e}$ [6]
			OR=1.26 (0.62, 2.57) $_{s}$ [2]	OR=1.57 (0.72, 3.41) $_{s}$ [2]
Severe obesity (ref=BMI <25)			**OR=6.16 (1.42, 26.66) $_{s, e}$ [6]**	**OR=8.19 (1.36, 49.13) $_{s, e}$ [6]**
Severe obesity (ref=BMI 25-34)		**OR=3.35 (1.43, 7.87) [8]**		**OR=4.06 (1.72, 9.57) $_{s}$ [8]**
Hypertension and heart disease				
Hypertension	**HR=1.58 (1.07, 2.32) $_{sm}$ [3]**	OR=1.82 (0.50, 6.63) $_{c}$ [9]	OR=0.7 (0.2, 2.0) $_{s, c}$ [10]	OR=0.81 (0.40, 1.64) $_{s}$ [2]
	OR=0.87 (0.50, 1.50) $_{s, e, c}$ [11]		OR=1.56 (0.83, 2.92) $_{s}$ [2]	OR=0.47 (0.13, 1.66) $_{s, e, c}$ [6]
			OR=0.79 (0.27, 2.28) $_{s, e, c}$ [6]	
Heart disease (incl prior MI or heart failure)	OR=0.72 (0.34, 1.50) $_{s, e, c}$ [11]	**HR=1.16 (1.08, 1.24) $_{s, c}$ [7]**	OR=1.52 (0.51, 4.51) $_{s, e, c}$ [6]	OR=0.70 (0.30, 1.64) $_{s}$ [2]
		OR=3.04 (0.45, 20.74) $_{c}$ [9]	**OR=4.2 (1.2, 14.2) $_{s, c}$ [10]**	**OR=3.41 (1.05, 11.06) $_{s, e, c}$ [6]**
			OR=1.07 (0.52, 2.23) $_{s}$ [2]	
Diabetes mellitus	**HR=1.59 (1.03, 2.45) $_{sm}$ [3]**	**HR=2.84 (1.01, 8.01) $_{c}$ [12]**	OR=1.91 (0.71, 5.19) $_{s, e, c}$ [6]	OR=2.13 (0.73, 6.22) $_{s, e, c}$ [6]

	OR=1.10 (0.60, 1.90) $_{s, e, c}$ [11]	HR=1.06 (0.99, 1.14) $_{s, c}$ [7]	OR=1.1 (0.3, 3.6) $_{s, c}$ [10]<br>OR=1.18 (0.64, 2.19) $_{s}$ [2]	 OR=0.94 (0.47, 1.89) $_{s}$ [2]
Cancer	**HR=3.50 (1.60, 7.64) $_{sm}$ [3]**<br>**HR=2.52 (1.66, 3.83) $_{sm, s, c}$ [14]**	**OR=2.45 (1.71, 3.50)* $_{s}$ [13]**<br>**HR=1.13 (1.02, 1.24) $_{s, c}$ [7]**		
COPD or other chronic respiratory disease	**HR=2.68 (1.42, 5.05) $_{sm}$ [3]**	**HR=1.17 (1.09, 1.27) $_{s, c}$ [7]**	OR=1.50 (0.47, 4.82) $_{s, e, c}$ [6]<br>OR=2.0 (0.2, 18.3) $_{s, c}$ [10]<br>OR=0.82 (0.40, 1.68) $_{s}$ [2]	OR=0.76 (0.20, 2.86) $_{s, e, c}$ [6]<br><br>OR=0.67 (0.29, 1.53) $_{s}$ [2]
Other comorbidities				
Neurological disorder, e.g. stroke		**HR=1.17 (1.06, 1.29) $_{s, c}$ [7]**		
Dementia		**HR=1.40 (1.28, 1.52) $_{s, c}$ [7]**		
Chronic kidney disease		**HR=1.28 (1.18, 1.39) $_{s, c}$ [7]**		
Liver disease		**HR=1.51 (1.21, 1.88) $_{s, c}$ [7]**		

ICU = Intensive care unit
COPD = Chronic obstructive pulmonary disease
HR=hazard ratio
OR=odds ratio
All estimates adjusted for age. Additionally adjusted for:
- sm = smoking
- s = sex
- c = other comorbidities
- e = ethnicity
- d = deprivation

Bold indicates estimates with a 95% confidence interval excluding the null value

* 95% CI not reported, but back-calculated from reported p value

List of included studies

* = pre-print article, not peer-reviewed

1. Nikpouraghdam, M., et al., Epidemiological characteristics of coronavirus disease 2019 (COVID-19) patients in Iran: A single center study. *Journal of Clinical Virology*, 2020. DOI: 10.1016/j.jcv.2020.104378

*2. Ebinger, J.E., et al., Pre-Existing traits associated with Covid-19 illness severity. medRxiv preprint, 2020. https://doi.org/10.1101/2020.04.29.20084533

3. Guan, W.J., et al., Comorbidity and its impact on 1590 patients with Covid-19 in China: A Nationwide Analysis. *European Respiratory Journal*, 2020. DOI: 10.1183/13993003.00547-2020

*4. Sapey, E., et al., Ethnicity and risk of death in patients hospitalised for COVID-19 infection: an observational cohort study in an urban catchment area. medRxiv preprint, 2020. https://doi.org/10.1101/2020.05.05.20092296

5. Cai, Q., et al., Obesity and COVID-19 severity in a designated hospital in Shenzhen, China. *Diabetes Care*, 2020. DOI: 10.2337/dc20-0576

6. Kalligeros, M., et al., Association of obesity with disease severity among patients with COVID-19. *Obesity*, 2020. DOI: 10.1002/oby.22859

7. Docherty, A.B., et al., Features of 20 133 UK patients in hospital with covid-19 using the ISARIC WHO Clinical Characterisation Protocol: prospective observational cohort study. *British Medical Journal*, 2020. DOI: 10.1136/bmj.m1985

8. Palaiodimos, L., et al., Severe obesity, increasing age and male sex are independently associated with worse in-hospital outcomes, and higher in-hospital mortality, in a cohort of patients with COVID-19 in the Bronx, New York. *Metabolism*, 2020. DOI: 10.1016/j.metabol.2020.154262

9. Wang, K., et al., Clinical and laboratory predictors of in-hospital mortality in patients with COVID-19: a cohort study in Wuhan, China. *Clinical Infectious Diseases*, 2020. DOI: 10.1093/cid/ciaa538

10. Yu, X., et al., Epidemiological and clinical characteristics of 333 confirmed cases with coronavirus disease 2019 in Shanghai, China. *Transboundary and Emerging Diseases*, 2020. DOI: 10.1111/tbed.13604

*11. Teo, J.T., et al., Impact of ethnicity on outcome of severe COVID-19 infection. Data from an ethnically diverse UK tertiary centre. medRxiv preprint, 2020. https://doi.org/10.1101/2020.05.02.20078642

*12. Zhang, Y., et al., Comorbid Diabetes Mellitus was Associated with Poorer Prognosis in Patients with COVID-19: A Retrospective Cohort Study. medRxiv preprint, 2020. https://doi.org/10.1101/2020.03.24.20042358

13. Mehta, V., et al., Case fatality rate of cancer patients with COVID-19 in a New York hospital system. *Cancer Discovery*, 2020. DOI: 10.1158/2159-8290.CD-20-0516

14. Dai, M., et al., Patients with Cancer Appear More Vulnerable to SARS-COV-2: A Multicenter Study during the COVID-19 Outbreak. *Cancer Discovery*, 2020. DOI: 10.1158/2159-8290.CD-20-0422
